# Visceral scalloping in pancreatic ascites: An uncommon manifestation of pancreatitis

**DOI:** 10.1515/pp-2020-0101

**Published:** 2020-02-26

**Authors:** Antriksh Kumar, Atul Rana, Anupam K Singh, Pankaj Gupta, Harjeet Singh, Vishal Sharma

**Affiliations:** Department of Gastroenterology, Postgraduate Institute of Medical Education and Research, Chandigarh, India; Department of Gastroenterology and General Surgery, Postgraduate Institute of Medical Education and Research, Chandigarh, India

**Keywords:** pancreatitis, peritoneal tuberculosis, peritoneal tumours, pseudomyxoma, scalloping

## Abstract

Scalloping of visceral organs like liver and spleen can cause certain peritoneal diseases. It has usually been described with pseudomyxoma peritonei and peritoneal carcinomatosis. Occasionally, it has also been described with certain benign conditions like peritoneal tuberculosis. We describe visceral scalloping in setting of pancreatic diseases. We believe that pancreatic fluid collections exert significant pressure on the visceral organs to result in scalloping of the visceral surfaces.

A 16-year-old male diagnosed as traumatic pancreatic ascites had ascitic fluid amylase levels of 60,000 U/L. Magnetic resonance imaging revealed ascites and scalloping of the liver surface (Figure 1A). Another 31-year-old male, chronic alcoholic, presented with alcohol-related acute pancreatitis. Computed tomography showed acute necrotic collection and scalloping of the liver margin (Figure 1B). Another, 54-year-old male presented with grade 4 traumatic pancreatitis. CECT revealed the presence of scalloped liver margin (Figure 1C). All these three patients were managed with percutaneous drains and one received pancreatic stenting with subsequent improvement.

**Figure 1:**
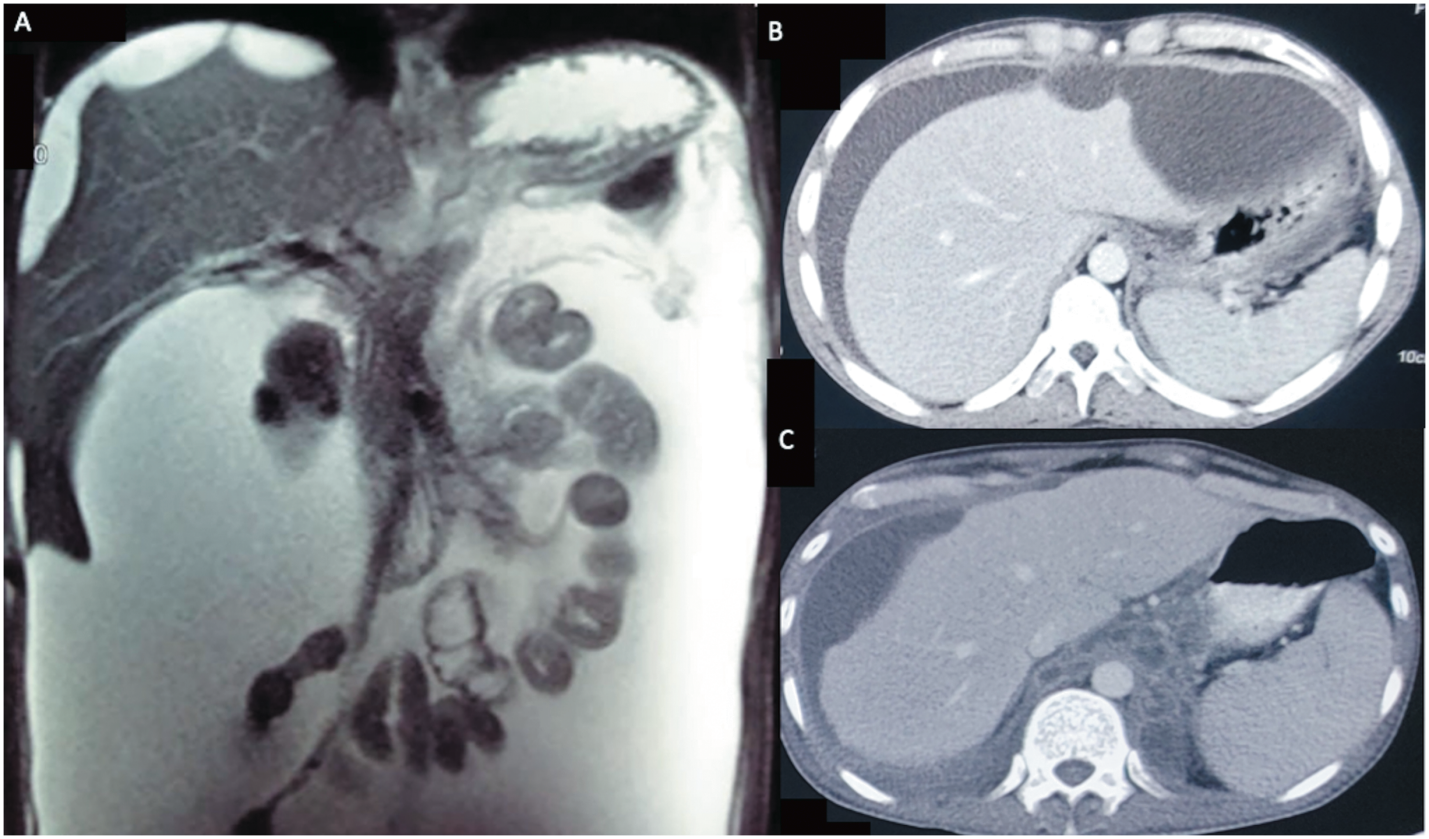
Depicting scalloping of the liver in pancreatitis.

(A) T2W MRI image showing massive ascites with scalloping of the liver surface. (B) Computed tomography showing scalloping of the left lobe of liver with ascites and collection. (C) Computed tomography showing ascites and scalloped liver surface.

The radiological finding of visceral scalloping, characteristic for pseudomyxoma peritonei and peritoneal carcinomatosis, has been occasionally reported with benign conditions [1, 2]. To the best of our literature search, it has never been described so far in the setting of acute or chronic pancreatitis. We postulate that indentation/extrinsic compression by pancreatic fluid collections can result in scalloping of viscera.

Informed consent was obtained from the patient/kin for the publication.

## References

[1] Seshul MB, Coulam CM. Pseudomyxoma peritonei: computed tomography and sonography. Am J Roentgenol 1981;136:803–6. 10.2214/ajr.136.4.803 6261563

[2] Sharma V, Bhatia A, Malik S, Singh N, Rana SS. Visceral scalloping on abdominal computed tomography due to abdominal tuberculosis. Ther Adv Infect Dis 2017;4:3–9. DOI:10.1177/2049936116685262. PMC5360295 28357060

